# Integrative Psychotherapy Works

**DOI:** 10.3389/fpsyg.2015.02021

**Published:** 2016-01-11

**Authors:** Cristina Zarbo, Giorgio A. Tasca, Francesco Cattafi, Angelo Compare

**Affiliations:** ^1^Department of Human and Social Science, University of BergamoBergamo, Italy; ^2^Department of Psychiatry, University of Ottawa, Ottawa HospitalOntario, ON, Canada; ^3^Department of Psychology, University of ChietiChieti, Italy

**Keywords:** integrative, psychotherapy, common factors, eclecticism, clinical psychology

## Integrative psychotherapy

A range of psychotherapy approaches have been recognized as effective and even the treatment of choice across the range of psychiatric diagnostic categories (e.g., see https://www.nice.org.uk/guidance/cg123 or https://www.nice.org.uk/guidance/conditions-and-diseases/mental-health-and-behavioural-conditions). However, in clinical practice, the choice of the most effective psychotherapy for each mental disorder is complicated by the existence of over 400 varieties of psychotherapy approaches that can be defined and classified in several ways according to their theoretical model (i.e., behavioral, systemic, cognitive, psychodynamic, etc.), format (i.e., individual, family, group), temporal length and frequency of the sessions, as well as any possible combination of these elements (Garfield and Bergin, 1994). Due to their different epistemologies and attempts to create rigid boundaries around the theories, dialog among these models has been limited.

In part to bridge this historical division, a number of leaders in the field have proposed an integrative approach to psychotherapy, which since the 1990s has been gaining wider acceptance (Norcross and Goldfried, 2005). For example, the Society for the Exploration of Psychotherapy Integration (SEPI; http://www.sepiweb.org/) is an international organization with a growing membership that includes some of the world's leaders in psychotherapy practice and research.

More commonly, psychotherapists choose one theoretical model and apply it in a flexible and integrative way in their therapy practice. In recent decades, an increasing number of psychotherapists do not prefer to identify themselves completely within a single approach, but prefer to define themselves as integrative or eclectic (Feixas and Botella, 2004). In a recent large survey of over 1000 psychotherapists, only 15% indicated that they used only one theoretical orientation in their practice, and the median number of theoretical orientations used in practice was four (Tasca et al., 2015). According to the integrative psychotherapy movement, a new research field is evolving toward the search for common goals, aiming at selecting theories and techniques among psychotherapy models and developing a new field in a collaborative and integrative manner. The integrative psychotherapy movement does not aim at combining all the psychotherapeutic models into one, but its purpose is to develop a new framework for dialog among different approaches (Feixas and Botella, 2004).

The term “integration” may denote different meanings. The so-called “integrative perspective” indicates a general flexible and inclusive attitude toward the different psychotherapeutic models (Greben, 2004). It aims to see what can be learned and introduced from various perspectives in practice. Integration in psychotherapy involves four possible approaches: theoretical integration (i.e., transcending diverse models by creating single but different approach), technical eclecticism (i.e., using effective ingredients from different approaches), assimilative integration (i.e., working primarily from within one model but integrating aspects of others when needed), and common factors approach (i.e., focusing on effective therapeutic practices that are common to all approaches; Kozarić-Kovacić, 2008; Castonguay et al., 2015).

## Why integrative psychotherapy works

### Integrative psychotherapy fits different patients, problems and contexts

There is a growing agreement among psychotherapists and researchers that no single psychotherapeutic approach can be effective and appropriate for all patients, problems, and contexts. Each existing psychotherapeutic model and approach is inadequate for some individuals (Norcross and Goldfried, 2005). Evidence-based research has demonstrated that psychotherapeutic treatments that are integrative in their nature (e.g., Interpersonal Psychotherapy, Schema Therapy, Cognitive analytic therapy) are effective for several psychiatric disorders (e.g., Depression, Post-partum depression, Social Anxiety disorders, Generalized Anxiety Disorders, Personality Disorders, Dissociative Identity) (Reay et al., 2003; Kellett, 2005; Hamidpour et al., 2011; Stangier et al., 2011; Masley et al., 2012; Roediger and Dieckmann, 2012; Clarke et al., 2013; Miniati et al., 2014).

### Integrative psychotherapy includes effective common factors

At the heart of psychotherapy integration is the important research findings that despite the varying theoretical rationales and approaches of different schools of psychotherapy, they produce similar outcomes (Barth et al., 2013). What has lead psychotherapists to integrate psychotherapy models is the evidence that common factors across psychotherapy approaches (e.g., therapeutic alliance, client expectations, therapist empathy, etc.) likely account for more outcome variance than the specific effects attributed to each psychotherapeutic approach (e.g., interpretations in dynamic therapies or cognitive restructuring in cognitive behavioral therapies) (Wampold and Imel, 2015). Specific therapeutic techniques contribute about 7% on the outcome variance in psychotherapy, while the common factors account for almost 20% of the outcome variance (Lambert and Bergin, 1992). In recent decades, clinicians and researchers have been coming to a growing consensus about the existence of common factors that are shared among several psychotherapeutic approaches (Norcross and Goldfried, 1992; Wampold and Imel, 2015). Common factors among psychotherapy approaches that have been associated with positive outcomes and therapeutic changes include: the ability of the therapist to inspire hope and to provide an alternative and more plausible view of the self and the world; the ability to give patients a corrective emotional experience that helps them to remedy the traumatic influence of his previous life experiences; the therapeutic alliance; positive change expectations; and beneficial therapist qualities, such as attention, empathy and positive regard (Stricker and Gold, 2001; Feixas and Botella, 2004; Norcross and Goldfried, 2005; Constantino et al., 2011; Horvath et al., 2011). Among the cited common factors, therapeutic alliance has the most evidence as a predictor of patient change (Feixas and Botella, 2004).

### Integrative psychotherapy is flexible to patients' needs and sensitive to therapeutic alliance

One key value of integrative psychotherapy is its individualized approach (Norcross and Goldfried, 2005). The integrative psychotherapy model aims to respond to the person, with particular attention to affective, behavioral, cognitive, and physiological levels of functioning, and to spiritual beliefs. Integrative psychotherapy allows for a better adaptation of the therapy to the distinctive characteristics and needs of each client, by allowing the therapist to tailor their knowledge of evidence-based treatments and approaches. The main emphasis of integrative psychotherapy is on the individual characteristics of the patient and on the therapeutic relationship, both considered as key elements of therapeutic change (Feixas and Botella, 2004), as well as on client motivation. This approach is in line with the recent guidelines by the American Psychological Association on what constitutes Evidence-Based Practice (American Psychological Association, 2006). In those guidelines, EBP are defined by research evidence, clinical judgment, and client factors. Consistent with this definition, integrative psychotherapy is not a technique applied to a passive patient, but the client is seen as an active participant in the therapy, and the therapist adjusts his or her approach depending on client characteristics and preferences. It is within the context of the therapeutic relationship that changes can be promoted and clients can most benefit from a caring and empathic therapist (Feixas and Botella, 2004).

## Be integrative, not eclectic

Psychotherapists commonly practice different types of psychotherapy integration, applying the common factors approach as well as assimilative integration or theoretical integration. The common factors approach tends to downplay the importance of specific effects or techniques of psychotherapies (i.e., two-chair technique, exposure, Socratic questioning, etc.), in favor of working with common factors known to be related to positive outcomes (e.g., therapeutic alliance, therapist empathy, client expectations, etc.; Norcross and Goldfried, 2005). On the other hand, assimilative integration involves working primarily from one theoretical approach (e.g., cognitive behavioral therapy) but also incorporating techniques from other psychotherapeutic approaches as needed for any given client or context (e.g., interpreting transference; Stricker and Gold, 2001). Finally, theoretical integration aims to bring together theoretical concepts from several different psychotherapeutic approaches and to develop a “Grand Unified Theory” of psychotherapy (Stricker and Gold, 2001).

The psychotherapy integration movement highlights that psychotherapy integration is not only the process of taking some techniques from various models and applying them as needed (i.e., technical eclecticism), but it involves also the focus on the link between theory, evidence, and technique (Norcross and Goldfried, 2005). In other words, integrative psychotherapy is different from technical eclecticism. An eclectic therapist chooses a technique because it may work or may be efficient, without concern for its theoretical basis or research evidence. If an eclectic psychotherapist's client experiences positive outcomes after receiving a specific technique, the therapist does not necessarily investigate why the positive change occurred in order to develop a generalizable model of treatment. In contrast, psychotherapy integration focuses on the relationship between an effective practice and its theoretical and empirical basis (Norcross and Goldfried, 2005). For example, evidence-based psychotherapy relationship practices (i.e., alliance, therapist empathy, congruence, positive regard, etc.), which are integrative in nature and based on common factors, have been the focus of a recent APA task force report (Norcross, 2011).

In conclusion, the evidence indicates that integration in general psychotherapeutic practice is desirable, even if clarification needs to emerge at the level of theory. In order to overcome this limitation of psychotherapy integration, psychotherapy orientations should cultivate integration and work closely together while maintaining their separate identities. International organizations like SEPI or the Society of Psychotherapy Research (http://www.psychotherapyresearch.org/) represent a good example of the possibilities of collaboration and integration among psychotherapists of different orientations. Moreover, a strong collaboration between integrationists and psychotherapy researchers could lead to the development of a unified background of knowledge and action that, in turn, will advance the promising integrative psychotherapy field (Castonguay et al., 2015).

## Author contributions

We declare that the manuscript has been seen and reviewed by all authors which have contributed to it in a meaningful way.

### Conflict of interest statement

The authors declare that the research was conducted in the absence of any commercial or financial relationships that could be construed as a potential conflict of interest.
